# Pigmented paraganglioma of the kidney: a case report

**DOI:** 10.1186/1746-1596-7-77

**Published:** 2012-06-28

**Authors:** Ling Zhao, Jie Luo, Honglei Zhang, Jiping Da

**Affiliations:** 1 Department of Pathology, China-Japan Friendship Hospital, Beijing, 100029, China

**Keywords:** Pigmented paraganglioma, Kidney, Differential diagnosis

## Abstract

Paragangliomas are rare neoplasms arising from undifferentiated cells of the primitive neural crest. We report a case of a 57-year-old patient with renal pigmented paraganglioma that was an incidental finding. Histopathological examination showed typical morphology of paraganglioma, as well as the unusual feature of large amounts of pigment in the cytoplasm of the tumor cells which was confirmed by bleached Fontana-Masson. Electron microscopy showed abundant, pleomorphic electron-dense granules consistent with neuromelanin. The tumor cells were positive for CD56 and chromogranin A, negative for HMB-45. The unique morphologic appearance represents divergent differentiation from neural crest. To our knowledge, the present case represents the first example of pigmented paraganglioma of the kidney.

**Virtual slides:**

The virtual slide(s) for this article can be found here: http://www.diagnosticpathology.diagnomx.eu/vs/2017147293711495.

## Background

Extra-adrenal paraganglia are specialized neural crest-derived cells that are scattered from the base of the skull down to the pelvic floor. Paragangliomas are tumors that arise from chromaffin cells at a variety of anatomic sites. Some of the unusual sites for paragangliomas include the kidney [1], uterus [2], and prostate [3]. Pigmented paragangliomas are very rare and less than thirteen cases of pigmented extra-adrenal paraganglioma have been reported in the English literature to date [2,4-9]. Our case is the first occurring in the kidney.

Herein a case of pigmented paraganglioma of the kidney in a 57-year-old man is described. The relevant differential diagnoses and probable histogenesis are discussed.

## Case presentation

### Clinical history

**Figure 1:**
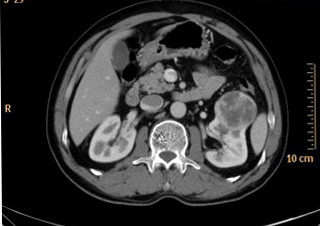
Contrast enhanced abdominal CT scan shows a well-delineated heterogeneous soft-tissue mass with small areas of calcification in the left kidney.

A 57-year-old man with an unremarkable medical history presented with an asymptomatic kidney mass that was discovered incidentally. Contrast-enhanced abdominal computed tomography (CT) scan showed a 4.5x4.5 cm heterogeneously enhanced mass with foci of calcification involving the left kidney (Figure 1). There were no signs of excess catecholamine secretion, and the blood pressure was 123/84 mmHg. Routine laboratory examinations were within normal limits, including the serum tumor markers. No clinical or imaging data suggested a paraganglioma, preoperative screening for catecholamines or metabolites was not performed. The left radical nephrectomy was performed. The right adrenal gland was normal. There was no evidence of recurrence or metastasis during the next twenty-two months of follow-up.

## Materials and methods

The specimen was fixed in a 10% neutral formalin solution and embedded in paraffin. Four micrometer-thick sections were prepared and stained with hematoxylin-eosin (H&E). Masson-Fontana stain (with and without pretreatment by potassium permanganate), Prussian blue, and periodic acid-Schiff (PAS) were also performed. Immunohistochemical staining was done on paraffin-embedded sections with a Polink-1 HRP detection system (Golden Bridge International Inc, Mukilteo, WA). Commercially available prediluted monoclonal antibodies were employed: Pan cytokeratin (Mouse mAb(AE1/AE3); 1:100), vimentin (Mouse mAb (V9); 1:100), CD10 (Mouse mAb(56 C6); 1:100), CD56 (Mouse mAb(56 C04); 1:100), S-100 protein (Mouse mAb(4 C4.9); 1:100), synaptophysin (Rabbit mAb(SP11); 1:100), HMB-45 (Melanosome) (Mouse mAb(HMB45); 1:100), chromogranin A (Rabbit mAb (SP12); 1:100), CK8/18 (Mouse mAb(Zym5.2); 1:100), CD34 (Mouse mAb(BI-3 C5); 1:100), Actin (Smooth muscle) (Mouse mAb(1A4); 1:100), and Ki-67 (Rabbit mAb(SP6); 1:200). All antibodies were obtained from Invitrogen Inc, Carlsbad, Calif except CD56, synaptophysin, chromogranin A, S-100 protein, and Ki-67 which were procured from Lab Vision, Cheshire, UK. The staining procedure followed manufacturer’s instruction. For electron microscopy examination, tissue was retrieved from the paraffin blocks, because glutaraldehyde-fixed material was not available.

### Pathological findings

Grossly, a solitary mass measuring 4.5x4x3.5 cm was located in the central region of the left kidney. The tumor was well demarcated from the adjacent kidney but not encapsulated. It exhibited solid, dark red to black appearance. Foci of hemorrhage were detected, while no necrotic area was observed. The tumor had no involvement with the left adrenal gland or with the renal vein.

**Figure 2:**
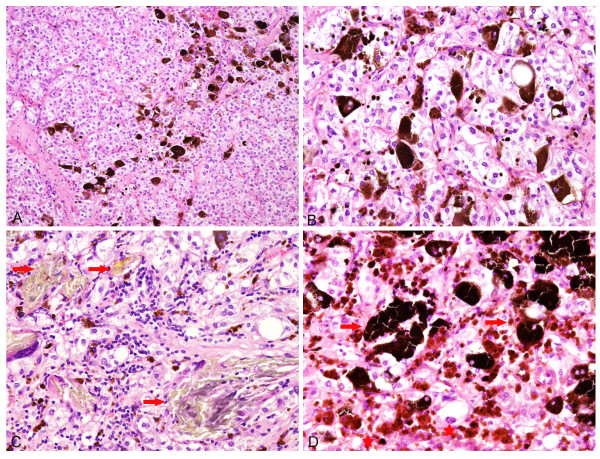
**The tumor cells are arranged in Zellballen pattern and have clear to finely granular cytoplasm (A, x 100; B, x 400).** Gamna-Gandy bodies are present (**C**, x 400, arrows). Abundant pigment granules are visible within the tumor cells (**D**, x 400). Note the coarse hemosiderin deposition (asterisks) and the finely granular pigment in the cytoplasm (arrows).

Microscopically, the tumor had the typical features of paraganglioma. The cells were characteristically arranged in nests (“Zellballen”) bound by a delicate fibrovascular stroma, having a mixture of alveolar and anastomosing trabecular patterns (Figure 2A). Hemorrhage within the tumor separated irregular islands of tumor cells. The tumor was composed of large round, or polygonal cells with ample clear to granular, pale eosinophilic cytoplasm. The nuclei were round, oval, hyperchromatic with conspicuous nucleoli (Figure 2B). Nuclear pseudoinclusions were occasionally noted. Stromal fibrosis with old hemorrhage was evident and extensive at the periphery of the tumor, with separation and distortion of nests of tumor cells. Gamna-Gandy bodies were present (Figure 2C). Neither necrosis nor mitotic figures could be observed.

**Figure 3:**
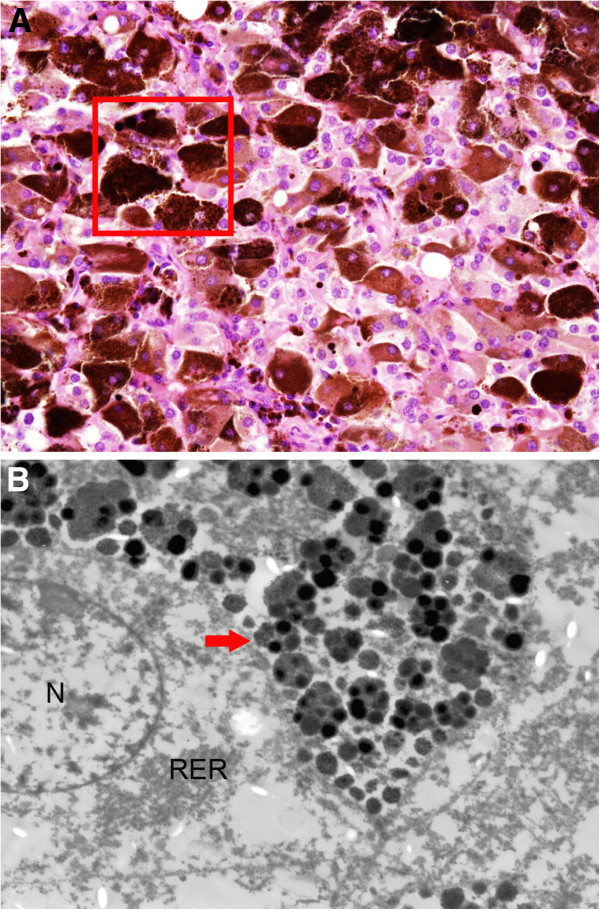
**The tumor contains abundant coarse pigment (A, x 200).** The area in the inset box in **A** is removed from paraffin blocks for ultrastructural analysis and is shown in **B**. Electron microscopy shows that a tumor cell has large, pleomorphic granules with varying electron density, size and shape, which are identified as neuromelanin (arrow). N, nucleolus; RER, rough endoplasmic reticulum (**B**, x 6000) (glutaraldehyde-refixed after retrieval from formalin-fixed material).

**Figure 4:**
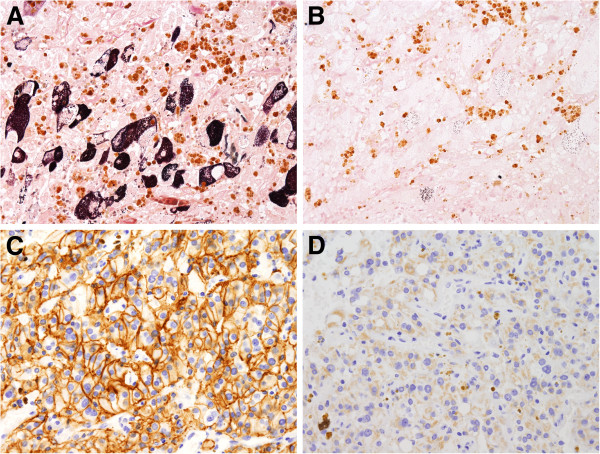
**The melanin pigment is positive for Masson-Fontana.** (**A**) and is bleached upon treatment with potassium permanganate (**B**). The tumor cells are strong staining for CD56 (**C**) and weak staining for chromogranin A (**D**).

Approximately two-thirds of the tumor cells showed some degree of pigmentation, ranging from finely dispersed small cytoplasmic granules to coarse material (Figure 2D). In some fields the pigment granules were so prominent as to obscure the true nature of the tumor (Figure 3A). In hemorrhagic areas, the pigment was coarse and refractile. Prussian blue stain showed abundant iron in cytoplasmic granules as well as extracellular aggregates. However, in other areas, the cytoplasmic pigment was finer and less refractile, which was negative for Prussian blue. The pigment was positive for Masson-Fontana and was susceptible to bleaching by potassium permanganate, consistent with neuromelanin (Figure 4A and B). The tumor cells were PAS negative.

Immunohistochemistry revealed that the tumor cells were highly positive for CD56 (Figure 4C) and showed weak expression of chromogranin A (Figure 4D). HMB-45 and synaptophysin were absent. The cells were negative for CK8/18, CD10, AE1/AE3, vimentin, and Actin. The Ki-67 labeling index was less than 1%. CD34 highlights the vascular supporting network around the cell nests. The tumor was devoid of S-100 protein positive cells.

Electron microscopy revealed a few heterogeneous granules of different densities with various sizes and configurations in the pigmented tumor cells which were interpreted as neuromelanin (Figure 3B). There were no melanosomes or premelanosomes.

These findings support a diagnosis of pigmented paraganglioma arising from the kidney.

## Discussion

Pigmented paragangliomas of adrenal medulla are unusual [10], and those arising in extra-adrenal paraganglia are rare. They are located in the uterus, retroperitoneum, lumbar spine, urinary bladder, orbit, heart, and mediastinum [2,4-9]. To the best of our knowledge, none have been described in the kidney.

The precise pathogenesis of renal paraganglioma is unknown. We postulate that the ectopic adrenal tissue or adrenal rests in kidney could be a plausible origin of the lesion. Another possibility is that paraganglioma may originate from the entrapped neuroendocrine progenitor cell of the dispersed neuroendocrine system resulting from aberrant migration from the neural crest during embryogenesis.

An unusual feature of this case was the presence of significant amounts of pigment. Based on histochemical staining or electron microscopy, pigment has been classified as neuromelanin, lipofuscin, or true melanin. Neuromelanin is found in neurons in the substantia nigra, locus ceruleus, and sympathetic ganglion cells as well as Parkinson’s disease. It has been regarded as a by-product of catecholamine synthesis in neurons and plays a neuroprotective role by binding toxic organic molecules, redox-active metal ions, and free radicals [11]. The production pathway is unclear.

Previous reports of pigmented paraganglioma have described the presence of neuromelanin [4,6]. The production of melanin was noted in Tavassoli’s report [2]. In later studies it has been shown that melanosomes and neuroendocrine granules are found in the same tumor cells [8]. Lipofuscin is found in tumor cells of retroperitoneal paraganglioma [12]. In our case, histochemical stains and electron microscopy showed that the pigment granules were true neuromelanin rather than lipofuscin. In addition, hemosiderin was also clearly seen in a minority of tumor cells. It seems most likely that the iron deposition arose from prior hemorrhage within the tumor.

The significance of the pigment and its mechanism of production are still obscure. Chromaffin cells, chief cells and melanocytes have the common embryological origin from the neural crest. The tumor arises by polyclonal evolution of a common neoplastic precursor cell which has melanocytic and paraganglionic characteristic within the same cells or show a mixed phenotypic expression. Tavassoli suggested that the close connection of the two biosynthetic pathways in producing either melanin or epinephrine might possibly account for melanogenesis in pigmented paraganglioma [2]. Hofmann et al. proposed that a genetic rather than an enzymatic regulation of the process was responsible for the simultaneous production of neuroendocrine granules and melanosomes [8]. Therefore, it is likely that there are other as-yet-undescribed mechanisms of tumorigenesis that are associated with pigmentation in paraganglioma.

The differential diagnoses include metastatic melanoma, metastatic paraganglioma and clear cell renal cell carcinoma (clear cell RCC). Paragangliomas have the characteristic nesting growth pattern, while malignant melanomas tend to have profound cellular pleomorphism and increased mitotic activity. Immunohistochemical profile is also helpful in distinguishing paraganglioma from malignant melanoma. In our case, the tumor cells were HMB-45 negative combined with positivity for chromogranin A and CD56, thus favoring paraganglioma rather than malignant melanoma. Further, negative history of primary melanocytic lesions of skin is of some value in establishing the correct diagnosis.

Because benign and malignant paragangliomas have the same histological appearance, and benign appearing tumors can result in metastatic disease to lymph nodes and other distant, non-paraganglia affiliated anatomic sites. The possibility of a metastatic paraganglioma must be considered into the differential diagnosis. However, the lack of clinical and radiographic support for a paraganglioma in another location, lack of capsular invasion, and lack of vascular invasion argue against a metastatic paraganglioma.

Another differential consideration is clear cell RCCs. Clear cell RCCs are composed of polygonal or cuboidal cells with clear or granular-eosinophilic cytoplasm. The pattern of growth is predominantly solid, alveolar and acinar, separated by fibrovascular septa, but may be admixed with cystic, papillary, tubular, and sarcomatoid patterns. Ronkainen et al. found CD56 positivity in 18% (23/128) of clear cell RCCs [13]. The morphologic and immunophenotypic features in common may lead to difficulty in distinguishing these entities. Nevertheless, paragangliomas lack the morphologic diversity which is seen in clear cell RCCs. Furthermore, immunohistochemistry may be helpful in differentiating clear cell RCC from paraganglioma. Clear cell RCCs are frequently immunoreactive with antibodies to CK8/18,vimentin, and CD10; this reactivity is not seen in paragangliomas.

Although varying combinations of capsular invasion, vascular invasion, necrosis, and mitotic activity have been seen in malignant paragangliomas, such features are commonly noted in tumors that have an indolent clinical course. None of them are found to be reliable to identify malignant biological behavior. A labeling index for Ki-67 may contribute to the differential diagnosis [14]. Due to the different scoring protocols with counts, the Ki-67 labeling in assessing malignancy is not widely accepted in clinical practice. The loss of S-100 protein has been reported to correlate with a more biologically aggressive clinical course for pheochromocytomas and paragangliomas [15]. In a study reported by Granger et al. [16], metastatic paragangliomas contained sustentacular cells in both the primary and metastatic lesions. The presence of sustentacular cells in the primary tumors could not be used as an absolute indicator of tumor metastatic potential. In our case, the sustentacular cells were absent. It should be noted that absence of sustentacular cells is not required for a diagnosis of malignancy. The presence of the pigment dosen’t appear to alter the biological behavior of paragangliomas. Distant metastases and invasion of adjacent organs are the only reliable indicators of malignancy. Although the presented tumor was extra-adrenal, the absence of local invasion and distance metastases showed the benign nature of this lesion.

Since there are no definite microscopic criteria for the distinction between benign and malignant tumors, complete surgical resection is the treatment of choice and careful follow-up is necessary.

In conclusion, pigmented paraganglioma is rare. We have described a pigmented paraganglioma which represents the first case of pigmented paraganglioma originating from the kidney and expands the morphologic spectrum of these unusual tumors.

## Consent

Written informed consent was obtained from the patient for publication of this Case Report and any accompanying images. A copy of the written consent is available for review by the Editor-in Chief of this journal.

## Competing interests

The authors declare that they have no competing interests.

## Authors’ contributions

LZ carried out pathological examination and wrote the manuscript. JL participated in pathological investigations. H-LZ carried out the immunohistochemical staining and collected the patient’s clinical information. J-PD participated in pathological investigations, revised manuscript for important intellectual content and had given final approval of the version to be published. All authors have read and approved the final manuscript.
